# APRIL is a novel clinical chemo-resistance biomarker in colorectal adenocarcinoma identified by gene expression profiling

**DOI:** 10.1186/1471-2407-9-434

**Published:** 2009-12-11

**Authors:** Russell D Petty, Leslie M Samuel, Graeme I Murray, Graham MacDonald, Terrence O'Kelly, Malcolm Loudon, Norman Binnie, Emad Aly, Aileen McKinlay, Weiguang Wang, Fiona Gilbert, Scot Semple, Elaina SR Collie-Duguid

**Affiliations:** 1 Section of Translational Medical Sciences, Division of Applied Medicine, School of Medicine and Dentistry, University of Aberdeen, Aberdeen, UK; 2 Departments of Oncology, Aberdeen Royal Infirmary, Aberdeen, UK; 3 Surgery, Aberdeen Royal Infirmary, Aberdeen, UK; 4 Clinical Radiology, Aberdeen Royal Infirmary, Aberdeen, UK; 5 University of Wolverhampton, Wolverhampton, UK

## Abstract

**Background:**

5-Fluorouracil(5FU) and oral analogues, such as capecitabine, remain one of the most useful agents for the treatment of colorectal adenocarcinoma. Low toxicity and convenience of administration facilitate use, however clinical resistance is a major limitation. Investigation has failed to fully explain the molecular mechanisms of resistance and no clinically useful predictive biomarkers for 5FU resistance have been identified. We investigated the molecular mechanisms of clinical 5FU resistance in colorectal adenocarcinoma patients in a prospective biomarker discovery project utilising gene expression profiling. The aim was to identify novel 5FU resistance mechanisms and qualify these as candidate biomarkers and therapeutic targets.

**Methods:**

Putative treatment specific gene expression changes were identified in a transcriptomics study of rectal adenocarcinomas, biopsied and profiled before and after pre-operative short-course radiotherapy or 5FU based chemo-radiotherapy, using microarrays. Tumour from untreated controls at diagnosis and resection identified treatment-independent gene expression changes. Candidate 5FU chemo-resistant genes were identified by comparison of gene expression data sets from these clinical specimens with gene expression signatures from our previous studies of colorectal cancer cell lines, where parental and daughter lines resistant to 5FU were compared. A colorectal adenocarcinoma tissue microarray (n = 234, resected tumours) was used as an independent set to qualify candidates thus identified.

**Results:**

APRIL/TNFSF13 mRNA was significantly upregulated following 5FU based concurrent chemo-radiotherapy and in 5FU resistant colorectal adenocarcinoma cell lines but not in radiotherapy alone treated colorectal adenocarcinomas. Consistent withAPRIL's known function as an autocrine or paracrine secreted molecule, stromal but not tumour cell protein expression by immunohistochemistry was correlated with poor prognosis (p = 0.019) in the independent set. Stratified analysis revealed that protein expression of APRIL in the tumour stroma is associated with survival in adjuvant 5FU treated patients only (n = 103, p < 0.001), and is independently predictive of lack of clinical benefit from adjuvant 5FU [HR 6.25 (95%CI 1.48-26.32), p = 0.013].

**Conclusions:**

A combined investigative model, analysing the transcriptional response in clinical tumour specimens and cancers cell lines, has identified APRIL, a novel chemo-resistance biomarker with independent predictive impact in 5FU-treated CRC patients, that may represent a target for novel therapeutics.

## Background

Significant progress has been made recently in the systemic treatment of colorectal adnocarcinoma (CRC). There are currently 8 agents licensed for use in the US and Europe 5-fluorouracil (5FU), floxuridine, capecitabine, irinotecan, oxaliplatin, cetuximab, panitumumab and bevacizumab [1]. Combination therapy is the standard of care for both early and advanced disease [1]. 5FU, or an oral analogue capecitabine, is a component of the majority of combination regimens and the low toxicity, ease and convenience of administration, favour its clinical use. However, a modest response rate due to clinical resistance to 5FU is a major limitation. Older studies with 5FU monotherapy demonstrate that the majority of CRC patients treated will not benefit from 5FU, for example the objective response rate to 5FU or capecitabine monotherapy in advanced CRC is 20% [1].

Identification of the clinically important mechanisms of resistance to 5FU would allow better selection of patients for 5FU therapy and the rationale design of targeted therapeutics to overcome resistance, and thus increase the proportion of patients deriving benefit from 5FU. A predictive biomarker for clinical 5FU resistance would clearly be useful, but progress has been limited in this area and investigation has thus far failed to fully explain the molecular mechanisms that areimportant for clinical 5FU resistance [2-4]. Preclinical and clinical studies have mainly focussed upon molecules concerned with 5FU metabolism (Dihydropyrimidine dehydrogenase (DPD), Thymidine phosphorylase (TP)) or Thymidylate Synthase (TS), a well characterised 5FU target [3,4]. Clinical studies in colorectal cancer, assessing these molecules by a variety of techniques (IHC, RT-PCR, ELISA, genotyping), while demonstrating correlation between benefit (such as response and survival) from 5FU or capecitabine, have so far failed either to demonstrate genuine clinical utility as predictive biomarkers or produce useful targeted agents [3]. Overall, given the widespread clinical use of 5FU or its oral formulations, there is still a need for novel discovery approaches in this area.

The global perspective provided by gene expression profiling has provided novel insights into the molecular mechanisms of clinical response to therapy in human cancers [5], although few studies have specifically addressed clinical therapy response in colorectal adenocarcinomas [6-10] and only 1 has analysed serial biopsies before and after treatment [8]. This report describes our prospectively designed discovery study, Aberdeen Microarray in Rectal Cancer Study-1 (AMRECS1) using a combined approach, identifying candidate molecules from clinical specimens and comparing them with our 5FU chemo-resistance data from cell line model systems [11]. We aimed to identify novel mechanisms of resistance to 5-fluorouracil (5FU) that are clinically relevant in CRC patients. Tumour biopsies were collected before and after pre-operative therapy in rectal cancer patients following staging and stratification with magnetic resonance imaging (MRI), to identify gene expression changes that occur following either 'short course' radiotherapy (SCRT) or 5FU-based concurrent chemo-radiotherapy (CRT). Gene expression profiles from these matched clinical specimens were compared with profiles generated from colorectal adenocarcinoma cell lines, both sensitive parental and derived daughter cell lines with increasing resistance to 5FU. Data is presented for the validation of one potential novel clinical 5FU resistance candidate APRIL/TNFSF13 in an independent set of 234 patients with colorectal cancer.

## Methods

### Patients, Follow up and Treatment

The study was approved by the North of Scotland Research Ethics Committee. Patients provided informed consent in accordance with the regulations and instructions of the North of Scotland Research Ethics Committee for study participation, including use and publication of results. Full clinicopathological details are provided in table 1 and 2 and in Additional File 1. Patients were selected for either SCRT or CRT based upon MRI staging features [12]. All the radiotherapy was CT planned, using a 3 field technique (posterior and two lateral fields), multileaf collimation and with patients having a full bladder during the radiotherapy. Surgery was performed either the following week, for SCRT patients, or 6 to 8 weeks after completion of chemo-radiotherapy.

**Table 1:** Locally advanced rectal adenocarcinoma patients analysed by gene expression microarray.

Patient	Treatment^1^	Stage at Diagnosis^2^	Diagnostic biopsy grade & histology	Diagnostic biopsy cellularity^3^	Surgical biopsy grade & histology	Surgical biopsy cellularity^3^	Pathological stage^4^
CRT1	CRT	T2N1 M0	moderately differentiated adenocarcinoma	60%	poorly differentiated adenocarcinoma	60%	T3N2
CRT2	CRT	T3N1 M0	moderately differentiated adenocarcinoma	60%	moderately differentiated adenocarcinoma	60%	T3N1
CRT3	CRT	T3N0 M0	moderately differentiated adenocarcinoma	60%	moderately differentiated adenocarcinoma	50%	T3N0
CRT4	CRT	T4N1 M0	moderately differentiated adenocarcinoma	50%	moderately differentiated adenocarcinoma	50%	T2N0
RT1	RT	T2N0 M0	moderately differentiated adenocarcinoma	60%	moderately differentiated adenocarcinoma	60%	T3N0
RT2	RT	T2N1 M0	moderately differentiated adenocarcinoma	60%	moderately differentiated adenocarcinoma	60%	T2N1
RT3	RT	T2N0 M0	moderately differentiated adenocarcinoma	50%	moderately differentiated adenocarcinoma	60%	T3N2
RT4	RT	T2N0 M0	moderately differentiated adenocarcinoma	60%	moderately differentiated adenocarcinoma	60%	T3N0
CON1	None	T3N1 M0	moderately differentiated adenocarcinoma	75%	moderately differentiated adenocarcinoma	70%	T3N1
CON2	None	T2N1 M0	moderately differentiated adenocarcinoma	50%	moderately differentiated adenocarcinoma	50%	T3N1

^1 ^CRT = neoadjuvant concurrent chemoradiotherapy; RT = Short course pre-operative radiotherapy. ^2 ^MRI and clinical stage. ^3 ^% Tumour versus normal cells in biopsy profiled. ^4 ^Pathological stage post-preoperative therapy

**Table 2:** Resected colorectal adenocarcinoma patients analysed by immunohistochemistry for APRIL protein expression on tissue microarray

Variable	Frequency/median(range)
**Age**	71 years (22-92)
***Gender***	
Male	121
Female	113
***Histological Grade***	
Poor	27
Moderate	199
Well	8
***Tumour site***	
Proximal colon	79
Distal colon	86
Rectum	69
***Stage***	
I	46
II	86
III (adjuvant chemotherapy)^1^	102 (63)
N2	48

^1^In this series 63/102 (62%) Stage III patients received adjuvant chemotherapy with 5FU

### Gene expression profiling

Tumour biopsies were collected at the time of endoscopic diagnosis of rectal adenocarcinoma and placed immediately into RNAlater (800 μl) (Ambion, Austin, Texas). Tumour biopsies collected at time of curative surgical resection were placed immediately into normal saline and a pathologist provided a representative tumour biopsy, which was placed immediately into RNAlater within 30 minutes (800 μl). Tissues were stored in RNALater at 4°C overnight (16-18 hours), then washed in 500 μl ice cold RNase free PBS (Ambion, Austin, TX) and snap frozen in liquid nitrogen. Long-term storage of tissues was at -80°C. Before RNA extraction, histological diagnosis and features were confirmed by frozen section histology. Extraction and purification of total RNA was performed using TRIZOL reagent (Invitrogen, Carlsbad, CA) and RNeasy Microkits (Qiagen, Venlo, The Netherlands), according to the manufacturer's instructions. Quantification of total RNA was performed by spectrophometry (260/280 ratio 1.9 to 2.2 for all samples). Quality of total RNA and cRNA was assessed using a BioAnalyser 2100 (Agilent technologies, Palo Alto, CA). Target preparation for the Affymetrix Genechips™ was according to manufacturer's instructions (Affymetrix, Santa Clara, CA). Specifically, 4 μg of total RNA was used for reverse transcription and synthesis and amplification of biotin labelled cRNA using the One cycle target labelling and control reagents. Clean-up of biotin-cRNA was performed with RNeasy Minikits (Qiagen, Venlo, The Netherlands). Fragmentation was performed using 20 μg of biotin-labelled cRNA. A hybridisation cocktail was prepared from 15 μg which was first hybridised to Test 3 GeneChips™ to assess sample quality (GAPDH 3':5' < 3 and Actin 3': 5' < 3) and then to HGU133 Plus2.0 GeneChips™ (10 μg) for gene expression analysis. Procedures for hybridisation, washing, staining and scanning of chips were carried out according to standard protocols (Affymetrix, Santa Clara, CA).

### Analysis of gene expression profiling data

Analysis of the gene expression data is described in detail in Additional file 2 and as described previously [11,13]. Raw data for gene expression is provided in MIAME complaint format in Array express, accession number E-MEXP-1901

### Immunohistochemistry

Description of the Tissue Microarray (TMA) is provided in previous publications [14]. A total of 268 colorectal tumours and 50 normal colon cores are represented, with 1 core per case. During the staining procedure 34 (13%) tumour cores were lost, leaving cores from 234 patients available for assessment. Antigen retrieval was performed by microwaving in 10 mM citrate (pH 6.0) for 20 minutes. An autostainer (Dakocytomation, Glostrup, Denmark) was used for staining the sections using a mouse monoclonal primary antibody for human APRIL/TNFSF13 (1:60 dilution, Abcam, Cambridge, UK) and Chemate-Envision detection system (Dakocytomation, Glostrup, Denmark), according to the manufacturer's instructions. All sections were double scored by 2 independent investigators who were blinded to the clinical data. Scoring discrepancies were resolved by examination of sections at a double-headed microscope. Sections were scored positive or negative for tumour and/or stromal staining. In addition tumour staining intensity was scored as weak, moderate or strong.

### Statistical analysis

Continuity corrected χ^2 ^test, with Fisher's exact test where appropriate, was used for binary categorical variables, Pearson's χ^2 ^test for non-binary categorical variables and Student's t-test for numerical variables. Kaplan-Meier curves were constructed to assess survival and the log rank test to assess statistical significance. The Cox proportional hazards model was used for multivariate analysis of survival. Two-sided *p *values of less than 0.05 were considered significant. All analyses were performed using SPSS for Windows, version 13.0 (SPSS Inc, Chicago, IL).

## Results

### Chemo-radiotherapy or radiotherapy altered gene expression in rectal cancer

In a pilot transcriptomics study of rectal cancer patients, we used oligonucleotide microarrays to profile the expression of over 47000 transcripts representing 38562 human genes in rectal tumour biopsies before and after pre-operative treatment with CRT (n = 4 patients); table 1). Rectal tumour biopsies before and after SCRT (n = 4 patients; table 1) were also analysed to enable comparison of gene expression changes in patients treated with 5FU-based chemo-radiotherapy with those observed in patients receiving radiotherapy alone. Rectal tumour biopsies, at diagnosis and surgical resection, from two patients who did not undergo any pre-operative treatment (table 1) were used to identify treatment-independent gene expression changes.

SOPs were developed and validated to allow collection of tissues at endoscopic diagnosis and at surgical resection, whilst preserving RNA integrity. Total RNA extracted from these tissues (10-30 mg) in this pilot study provided sufficient yield (8 to 40 ug) and quality total RNA for gene expression analysis on Affymetrix oligonucleotide microarrays. Raw gene expression data is provided in MIAME complaint format in Array express, accession number E-MEXP-1901.

**Figure 1:**
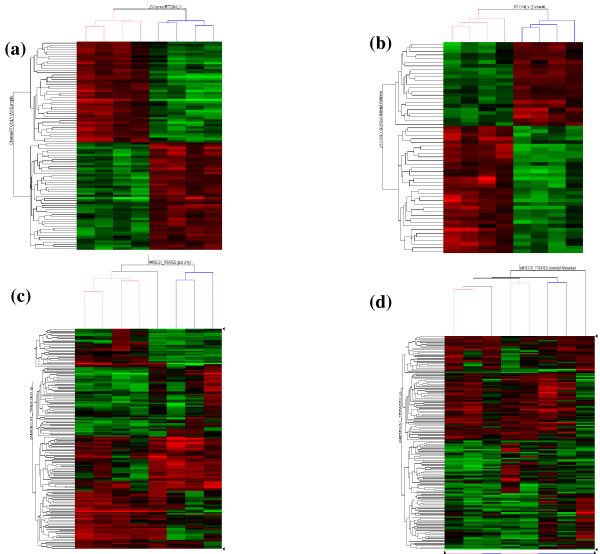
**Hierarchical cluster analysis of chemoradiotherapy or radiotherapy treated tumours**. This analysis separates pre- and post-treatment biopsies using (a) 86 genes identified as changed in chemoradiotherapy treated patients and (b) 51 genes identified as changed in short course radiotherapy treated patients. (c) Post-treatment tumour biopsies, cluster according to treatment received with the combined set of 137 genes, but (d) pre-treatment tumour biopsies do not. Columns represent tumour samples and rows represent genes (red: up-regulated and green: down-regulated, radiotherapy [blue] or chemoradiotherapy [pink]).

Threshold and probabilistic filtering of the data (see Additional file 2) identified 86 genes (91 probe sets) consistently, significantly and specifically altered following 5FU-based CRT and 51 genes (58 probe sets) following SCRT (see *Additional File *3 for details of genes and fold change following therapy). Hierarchical cluster analysis, highlights 2 distinct clusters of genes up-regulated or down-regulated following CRT (figure 1A) or SCRT (figure 1B). The expression profiles of each of these gene sets clearly separates pre- and post-treatment samples into two primary clusters for each treatment group (figure 1). A matrix analysis (DMTv1.0, Affymetrix, CA) of therapy-altered gene sets identified using threshold filtering alone (see *Additional file *2; 697 probe sets in CRT group and 570 in SCRT group, including 86 overlapping), reveals that these genes sets are significantly non-overlapping (p = 0.010), demonstrating highly distinct alterations to the tumour transcriptome following treatment with SCRT or 5FU-based CRT.

The biological functions of the CRT and SCRT altered gene sets were evaluated (additional file 4). While many of the same key biological pathways are identified in each treatment group, consistent with a co-ordinated transcriptional response, there are some pathways only altered following CRT and some pathways (cell death and cell cycle) where there is numerically significantly more change in gene expression in the CRT treated patients (additional file 4).

This represents an initial pilot study of the first samples in our rectal cancer patient cohort. It is important to note that the small sample size, necessitates validation of these candidate gene expression changes in a larger cohort. The primary aim of this study was to identify candidate 5FU resistance markers in rectal tumours, in a pilot discovery study using a transcriptome-wide approach and to validate key candidate/s that may have mechanistic relevance in a larger cohort. Identification and validation of one such marker is described below.

### APRIL/TNFSF13 in colorectal cancer

As we were interested in potential mediators of 5FU resistance in rectal tumours *in vivo*, we further mined the gene expression analysis using a pathway focussed analysis of cell deaths pathways, including those involved in regulation or execution of caspase-dependent apoptotic, caspase-independent and necrotic cell death genes (n = 2177 genes; additional file 5). Threshold and probabilistic filtering of the gene expression data identified 17 cell death genes consistently and significantly altered in rectal tumours following chemo-radiotherapy (additional file 6). Several of these genes have been implicated in colorectal cancer pathogenesis and the pathogenesis of other cancers, and also radioresistance, but none previously in 5FU chemoresistance (for more details see additional file 6). Comparison of the 17 cell death genes altered in response to 5FU based CRT in tumours from rectal cancer patients, with gene expression changes identified in our previous study of 5FU resistant cancer cell lines [11], demonstrated 4 of the 17 genes up-regulated following CRT (but not radiotherapy alone) in rectal cancer patients and in 5FU-resistant cancer cells compared to the sensitive parental lines(See additional file 6, Table S6.1). This included the TNF superfamily ligand, APRIL (TNFSF13).

APRIL has been characterised as promoting cell survival and cell proliferation and this involves NFκB activation [15-19]. In addition, APRIL mRNA has been shown to be increased in colorectal tumours compared to normal mucosa [17]. These data supported further investigation of a putative functional role for APRIL in clinical 5FU chemo-resistance.

**Figure 2:**
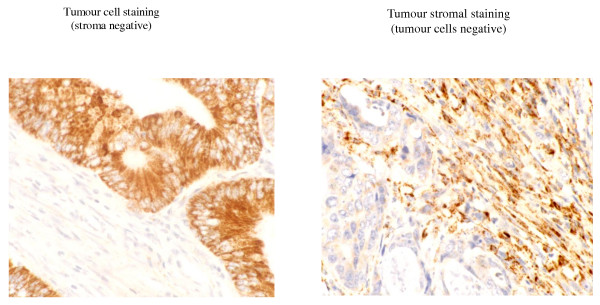
**Immunohistochemistry for APRIL in resected colorectal adenocarcinomas**. Staining for APRIL was seen in the tumour cells (membrane and cytosol) and stroma (extracellular matrix and stromal cells) of colorectal adenocarcinomas. All combinations of tumour cell and stromal staining were seen. Tumour cell staining could be scored weak, moderate and strong. Examples show strong tumour cell staining and stromal staining.

APRIL protein expression was evaluated in 234 resected colorectal adenocarcinomas and 50 normal colon or rectal mucosa specimens (table 2). APRIL protein was not expressed in normal colon tissues but was, as expected, expressed in both colorectal tumour cells and the tumour stroma (Table 3 and figure 2). Tumour cell staining was observed in the cytosol and membrane of tumour cells (figure 2). Stromal staining was evident in both the extracellular matrix and also in stromal cells (figure 2).

**Table 3:** Tumour cell and stromal expression of APRIL protein in colorectal adenocarcinomas.

APRIL Immunohistochemistry		
**Tumour Cell**	Positive	130 (55.6%)
	*Weak*	*70 (29.9%)*
	*Moderate*	*49 (20.9%)*
	*Strong*	*11 (4.7%)*

**Stroma**	Negative	104 (44.4%)
	Positive	121 (51.7%)
	Negative	113 (48.3%)

Immunohistochemical analysis of a rectal adenocarcinoma tissue microarray (n = 234 tumours) demonstrated that APRIL protein was expressed in tumour cells and/or tumour stroma. Positive stromal expression was strong. In tumour cells expressing APRIL, intensity was weak, moderate or strong. The number of rectal adenocarcinomas with positive staining for APRIL protein. Percentage of the total (n = 234) is in parentheses. There was a significant correlation between tumour cell and stromal expression (p = 0.048). There was no significant association between tumour cell or stromal staining and age, gender, histological grade, tumour site or Duke's stage (all p > 0.20. Data not shown).

### APRIL, a putative 5FU chemo-resistance factor and predictive biomarkerin 5FU treated colorectal cancer patients

**Figure 3:**
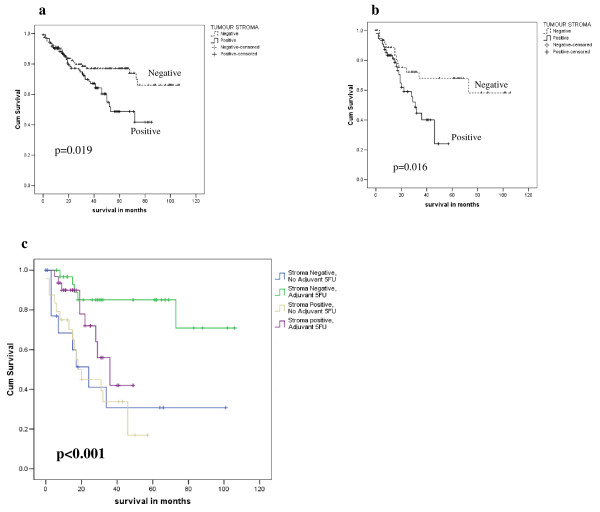
**APRIL protein expression in tumour stroma and survival of colorectal cancer patients**. (a). Kaplan-Meier survival plots for tumour stroma APRIL protein expression analysed by immunohistochemistry of 234 colorectal cancer patients following surgical resection.(b) Stromal staining for APRIL in Stage III patients following surgical resection (n = 102) (c) Combined analysis of stage III patients (n = 102) stratified according to adjuvant therapy and tumour stroma APRIL protein. P value is log rank test.

We examined the relationship between APRIL protein expression and survival after surgical resection. We prospectively determined that we would evaluate both tumour cell and tumour stromal expression of APRIL protein due to its characterized biological function as a secreted autocrine and/or paracrine molecule. There was no significant relationship between APRIL protein expression in tumour cells and survival (Additional file 7). In contrast, expression of APRIL protein in the tumour stroma was associated with poor survival (n = 234, p = 0.019, figure 3a), including in stage III patients (n = 102, p = 0.016, figure 3b), but was not associated with survival in Stage I or II (n = 46 p = 0.601 and n = 86 p = 0.440, respectively, Additional File 7).

In light of our hypothesised role of APRIL in 5FU resistance, we stratified the Stage III patients according to whether or not they received adjuvant chemotherapy with 5FU following surgical resection of their primary tumour. Stage I and II patients did not receive adjuvant chemotherapy in this series. Tumour stroma expression of APRIL protein is only associated with worse survival in those patients treated with adjuvant 5FU and there is no relationship with survival in Stage III patients not treated with adjuvant chemotherapy (n = 102, p < 0.001, figure 3c). In 5FU treated Stage III patients (n = 63), median survival for stroma positive is 36 months with predicted 5 year survival 42.0% (95% confidence interval 11.8% - 72.2%); median survival not yet reached for stroma negative and predicted 5 year survival is 85% (95% confidence intervals 71.7%-98.6%). Multivariate analysis confirms expression of APRIL protein in the tumour stroma as an independent prognostic factor in chemotherapy treated Stage III patients, with a HR of 6.25 (95% CI 1.48-26.32, p = 0.013, table 4).

**Table 4:** Multivariate analysis using Cox proportional hazards regression model for adjuvant chemotherapy treated Stage III patients.

Variable	HR	95% Confidence Interval	p value
Age (>70 vs <70)	1.006	0.955-1.059	0.835
Gender (Female vs Male)	0.532	0.159-1.783	0.307
Grade (poor vs moderate vs well)	N/A	N/A	0.873
Site (Proximal vs Distal)	5.015	0.695-36.191	0.110
**APRIL** **Tumour Stroma Staining** **(positive vs negative)**	**6.250**	**1.471-26.316**	**0.013**
APRIL Tumour Cell Staining (positive vs negative)	0.64	0.200-2.044	0.452

The survival of the 5FU treated Stage III colorectal cancer patients who express APRIL protein in the tumour stroma parallels survival observed in Stage III patients who did not receive adjuvant therapy (treatment decision due to patient or physician preference), irrespective of APRIL protein expression (figure 3c). In contrast, the APRIL negative patients have an excellent predicted 5 year survival and have a clear and statistically significant (p < 0.001) survival benefit compared to untreated or APRIL positive 5FU treated patients (figure 3c). These data suggest that APRIL has no prognostic impact in colorectal cancer treated by surgical resection alone, but has predictive impact for benefit from adjuvant 5FU in colorectal cancer patients.

## Discussion

Global gene expression profiling of clinical response to therapy has provided a useful means for biomarker and novel target discovery in several solid tumours [5,13]. The work described in this paper has used and extended this experimental approach to rectal adenocarcinomas. The data presented constitutes an analysis from gene expression profiling of prospectively collected pre- and post-treatment tumour specimens from patients with rectal adenocarcinomas receiving pre-operative therapy.

Since a small number of rectal adenocarcinomas have been profiled (n = 10), stringent and focussed analysis of the microarray data was applied to identify leads for further investigation. This included hypothesis-driven focus on cell death pathways and comparison with our previously published cell line work. The key candidate was subsequently validated a in larger independent set (n = 234) using a different technique (immunohistochemistry).

The biological validity of the experimental model and the data is confirmed by the finding of significant alterations in the gene expression of previously implicated molecules and pathways, for example p21 which has been implicated in numerous studies [20-25]. The biological pathways identified (information 3 and 4) suggest a co-ordinated transcriptional response to radiotherapy- and CRT- induced cellular stress, consistent with other reports involving gene expression profiling in cell lines and several different cancer types [2,11,13,25-29]. We hypothesize that this reflects distinct biological effects of these two treatments. However, the possibility of effects due to time course differences in the tumour sampling in each group cannot be excluded.

A supervised analysis of cell death genes, reveals shared genes and pathways. The analysis supports the hypothesise that initiation of cell death is a common final pathway resulting from a multitude of upstream responses to the insult and resultant cellular stress of cytotoxic chemotherapy or radiotherapy thereby accounting for gene expression overlap seen.

The majority of the genes identified in our analysis represent genes and pathways that have not previously been implicated in clinical response of rectal adenocarcinoma or as mechanisms of action or resistance to radiotherapy or 5FU or 5FU-based CRT. This is consistent with the findings of other gene expression profiling studies in rectal adenocarcinoma or other tumour types for radiotherapy or 5FU [6,8-11,26,28-30]. However, it is important to note that this discovery phase utilised a small sample cohort and the candidate gene expression changes require further validation in a lrger independent cohort.

APRIL/TNFSF13 was found to be upregulated following CRT but not radiotherapy alone in rectal cancers and was also up-regulated in 5FU resistant cell lines in our previous studies [11]. The biological function of APRIL as a secreted molecule that has autocrine and paracrine functions to promote cell survival and proliferation and its previously documented expression in colorectal adenocarcinoma but not normal cells outside the immune system, supported it's further investigation as a novel mechanism of 5FU action and resistance, and as a predictive biomarker [15-19,31-35].

This study found that expression of APRIL protein in colorectal tumour stroma was associated with worse survival, but only in those patient's treated with adjuvant 5FU chemotherapy. This relationship was also maintained in a multivariate analysis of 5FU chemotherapy treated Stage III colorectal adenocarcinoma patients (HR 6.25, 1.47-26.31, p = 0.013), in which the Hazard ratio compares favourably to other previously published putative 5FU predictive biomarkers in colorectal cancer [2-4]. Tumour cell expression of APRIL was correlated with stromal staining but was not significantly associated with survival. Overall, APRIL appears to have no therapy independent prognostic impact in colorectal adenocarcinoma in this analysis.

Within the limitations of a retrospective study, these results suggest that APRIL may have clinical utility as a predictive biomarker to select patients who would not benefit from adjuvant 5FU monotherapy. For example, currently adjuvant 5FU is used clinically in an empirical way without predictive biomarkers in stage III patients and in this paradigm the majority of patients with Stage III cancers will not benefit from 5FU. Therefore, the ability to identify some of these stage III patients who will not benefit from 5FU has clear potential clinical utility in optimising and individualising clinical use of 5FU in this setting. An important question is whether APRIL confers cross resistance to other active agents used to treat colorectal cancer, especially Oxaliplatin and Irinotecan, this would be potentially useful to guide 5FU combination adjuvant therapy in stage III patients, but especially in stage II patients where 5FU alone appears to have limited benefit.

The data allows us to hypothesise that APRIL may provide a useful novel therapeutic target. Morphological examination has suggested that positively staining stromal cells include lymphocytes and fibroblasts, but not endothelial cells. This is consistent with evidence indicating that APRIL is predominantly secreted and exerts it's effects via cell surface receptors, acting in a paracrine or autocrine fashion [15-19,31-35].

Our data indicate that APRIL might be secreted by tumour cells or stromal cells within the tumour. The APRIL signalling mechanisms that may mediate tumour cell survival are not well characterised [32]. However, in *vitro *work in glioma cell lines and *ex vivo *studies in BCLL, has shown that APRIL stimulates proliferation and inhibits apoptosis in response to a wide range of stimuli, including CD95L, TRAIL and cytotoxic drugs and survival in B-CLL cells involves NFκB activation [15-19,31-34]. More recently it has been suggested that tumour infiltrating neutrophils may be an important source of APRIL production in solid tumours [35].

If APRIL is functional as an extracellular secreted molecule this makes it amenable to targeting with either a small molecule inhibitor or monoclonal antibody, as has been employed successfully for other targets in solid tumours e.g. bevacizumab against VEGF. An anti-APRIL targeted therapy may be useful in reversal of acquired 5FU resistance or in combination in patients whose tumours over-express the molecule.

The lack of therapy independent prognostic impact suggests that an anti-APRIL therapy may not have anticancer activity on it's own, but the cell survivalpromoting activity may be more generally applicable to other therapeutic cell death stresses. Therefore, combination of an anti-APRIL agent with agents other than 5FU may be active, and our cell line data also suggest that they may be active in other tumour types, such as breast cancer.

## Conclusions

In this study we have used a combined investigative model, analysing the transcriptional response in clinical tumour specimens from rectal adenocarcinomas and cancer cell lines, to identify APRIL, as a novel 5FU chemo-resistance biomarker. We have validated its importance in an independent set of colorectal adenocarcinomas. This data supports further investigation of the clinical utility of APRIL as a predictive biomarker for 5FU resistance in colorectal adenocarcinomas and other solid tumour types and also as a target for novel therapeutics aimed at reversal of clinical resistance to 5FU and its oral analogues.

## Competing interests

The authors declare that they have no competing interests.

## Authors' contributions

RDP designed the study, completed ethical submission, consented patients for study, performed, analysed and interpreted gene expression profiling and immunohistochemistry and wrote the manuscript. LMS participated in study design, ethical submission, consenting of patients for study, interpretation of immunohistochemical data, and writing of manuscript GIM- participated in study design, histopathological review of specimens, provision of tissue microarray, analysis and interpretation of immunohistochemical data, and writing of manuscript. GMac- participated in study design, and consenting of patients for the study. T O'K, NB, EA, AMc- Consented patients for study, and provided fresh tumour tissue for gene expression profiling. WW- Assisted with analysis of gene expression data. FG- participated in study design, performed MRIs and reported MRIs SS- participated in study design and reporting of MRIs. ECD - participated in study design, assisted with ethical submission, assisted with analysis and interpretation of gene expression and immunohistochemical data, and writing of manuscript. All authors read and approved the final manuscript.

## Pre-publication history

The pre-publication history for this paper can be accessed here:

http://www.biomedcentral.com/1471-2407/9/434/prepub

## Supplementary Material

Additional file 1 **Further details of Patients and Treatments**. Clinicopathological, selection criteria, staging and chemotherapy and radiotherapy protocol details for patients in the study. Click here for file

Additional file 2 **Details of analysis of Gene Expression Profiling Data**. Details of quality control, normalisation and analysis for identification of genes whose expression is consistently and significantly altered as a consequence of chemoradiotherapy or radiotherapy. **Figure S2**. Schematic to illustrate bioinformatics analysis performed to identify genes whose expression was consistently and significantly altered as a result of either neoadjuvant chemoradiotherapy or short course radiotherapy. Click here for file

Additional file 3 **Details of genes identified in analysis of rectal adenocarcinomas**. Details of genes identified in analysis of rectal adenocarcinomas whose expression is consistently and significantly changed after treatment with chemoradiotherapy or radiotherapy. **Table S3.1-**. List of 86 genes (91 probe sets) whose expression is consistently and significantly changed after treatment with chemoradiotherapy. **Table S3.2 -**List of 52 genes (58 probe sets) whose expression is consistently and significantly chaged after treatment with short course radiotherapy. Click here for file

Additional file 4 **Biological pathways altered following neoadjuvant radiotherapy or chemoradiotherapy in rectal tumours**. The number of genes in each biological pathway whose expression was altered following chemoradiotherapy or radiotherapy is shown. Gene ontologies (biological function) were assigned according to GO, Genespring v6.1, Netaffx, EntrezGene, RefSeq and literature searches using Medline and ISI. **Table S4 **- The number of genes in each biological pathway whose expression was altered following chemoradiotherapy or radiotherapy. Click here for file

Additional file 5 **Cell death gene list used for supervised gene expression analysis**. Xcel file with list of identified 2177 genes involved in the control, regulation and execution of cell death (apoptotic and non-apoptotic forms) that were represented on the HGU133 Plus 2.0 GeneGhip, using databases (GO, Genespring v6.1, RefSeq, EntrezGene) and literature searches (Medline and ISI). Click here for file

Additional file 6 **Details of Supervised analysis of Cell Death Pathways**. Schematic representations to explain supervised bionformatic analysis of cell death pathways. **Figure S6 **Schematic illustrating the bioinformatics analysis performed for the supervised analysis of cell death genes. GCOSv1.2 and Genespring v6.1 were used for the analyses. **Table S6**. List of Cell death genes identified in this analysis in CRT and SCRT treated rectal adenocarcinoma patients. **Table S6.1**. Genes identified as candidate novel mechanisms of 5FU chemoresistance or sensitivity from gene expression profiling experiments of 5FU resistant colorectal and breast cancer cell lines Click here for file

Additional file 7 **Additional survival analyses for APRIL protein expression in colorectal adenocarcinomas**. Kaplan-Meier survival plots for APRIL protein expression in tumour cells of colorectal adenocarcinom patients in stage I, II and II and APRIl stroma expression in Stage I and II **Figure S7.1 **Kaplan-Meier survival plots for APRIL immunohistochemistry showing no significant relationship for tumour cell protein expression and survival. All patients (n = 234), analysed according to intensity of APRIL staining in tumour cells [weak, moderate or strong (b)] or positive versus negative tumour cell staining (a), or stratified according to stage Dukes A/Stage I, Dukes B/Stage II and Dukes C/Stage III (c). **Figure S7.2**. Kaplan-Meier survival plots for APRIL immuno-histochemistry showing that positive staining in the tumour stroma shows no association with survival in Duke's A/Stage I (n = 46) or B/Stage II tumours (n = 86). Click here for file
