# Supplementary material for: APRIL is a novel clinical chemo-resistance biomarker in colorectal adenocarcinoma identified by gene expression profiling
Source: BMC Cancer. 2009 Dec 11;9:434. doi: 10.1186/1471-2407-9-434 (PMC2801520; doi:10.1186/1471-2407-9-434)
Supplement: Additional file 1 — Further details of Patients and Treatments. Clinicopathological, selection criteria, staging and chemotherapy and radiotherapy protocol details for patients in the study. [file 1471-2407-9-434-S1.DOC]

Additional File- 1

Further details of Patients and Treatments

The study was performed with the approval of the regional ethics committee and within their published guidelines. Patients were recruited prospectively between March and October 2004 and were eligible if they had a histological diagnosis of locoregional rectal adenocarcinoma, were > 18years of age, were able to give written informed consent and were medically fit for operative treatment and /or neoadjuvant concurrent chemoradiotherapy (WHO PS 0-2, no significant medical co-morbidities in particular no history of unstable or severe ischemic heart disease, and adequate renal (creatinine clearance >50ml/min) and liver function (Billirubin <1.5 ULN, transaminases and alkaline phosphatase <2x ULN ) Staging was with , clinical examination (examination under anaesthesia at the discretion of the treating surgeon) high resolution MRI of the pelvis, and CT scan of the thorax and abdomen. Clinical stage was according to TMN classification of UICC 6^th^ edition 2002. Patients were selected for neoadjuvant concurrent chemoradiotherapy based upon clinical and MRI staging features (clinically fixed tumours, T4 tumours, T3 tumours below the levator ani, N2 tumours, and tumours extending to within 1mm of mesorectal fascia). Patients were similarly selected for short course neoadjuvant radiotherapy based upon clinical and MRI staging features (distal rectal tumours with no adverse MRI or clinical features as described for chemoradiotherapy selected patients). Patients recruited to this study receiving neoadjuvant treatment represent a consecutive series of 4 patients treated with neoadjuvant chemoradiotherapy and 4 patients treated with short course radiotherapy analysed as part of a planned interim analysis in the pilot phase of the project. The control group of two patients were selected from patients presenting parallel to those receiving neoadjuvant treatment. Regimens were as follows; neoadjuvant chemoradiotherapy: Folinic acid 20mg/m^2^ 5FU 350mg/m^2^ d1-5 and d29-33 with 45 Gy in 25 fractions; and short course radiotherapy 25Gy in 5 fractions. All radiotherapy was CT planned. Patients proceeded to surgical resection 4-6 weeks after completing neoadjuvant chemoradiotherapy and 1 week after completion of neoadjuvant short course radiotherapy. The surgical procedure performed was at the discretion of the treating surgeon. Pathological stage was recorded according to TNM classification of the UICC 6^th^ edition 2002.

All radiotherapy was CT planned. Patients with pathological stage III or high risk Stage II (defined as pT4 tumours, mucinous tumours or presence of extramural vascular invasion) were offered adjuvant chemotherapy with one of the following 5FU regimens: Modified de Gramont ( Folinic Acid 200mg/m2 over 2 hours followed by 5FU bolus 400mg/m2 followed by continuous infusion 600mg/m2 over 22 hours given on days 1 and 2 of a 14 day cycle for 6 cycles) or QUASAR weekly ( weekly bolus infusions of 5FU 370mg/m2 and folinic acid 175mg for 24 weeks).
