# Supplementary material for: APRIL is a novel clinical chemo-resistance biomarker in colorectal adenocarcinoma identified by gene expression profiling
Source: BMC Cancer. 2009 Dec 11;9:434. doi: 10.1186/1471-2407-9-434 (PMC2801520; doi:10.1186/1471-2407-9-434)
Supplement: Additional file 2 — Details of analysis of Gene Expression Profiling Data. Details of quality control, normalisation and analysis for identification of genes whose expression is consistently and significantly altered as a consequence of chemoradiotherapy or radiotherapy. Figure S2. Schematic to illustrate bioinformatics analysis performed to identify genes whose expression was consistently and significantly altered as a result of either neoadjuvant chemoradiotherapy or short course radiotherapy. [file 1471-2407-9-434-S2.DOC]

## Additional File-2

## Analysis of Gene Expression Profiling Data

## Quality Control and Normalisation: Initial quality control analysis and normalisation of data was performed using GCOSv1.2 (Affymetrix, Santa Clara, California). The gene expression signals were normalised using scaling of all probe sets to an arbitrary target signal of 100 (GCOSv1.2). This data was utilised for QC and generation of ‘detection call’ and ‘change call’ gene lists (MicroDBv3.0 and DMTv3.0, Affymetrix). Further details of these algorithms have been described previously [Petty et al 20006, Wang et al., 2004- see manuscript for references] and are available from the software provider ([***http://www.affymetrix.com/support/downloads/manuals/data_analysis_fundamentals_manual.pdf***](http://www.affymetrix.com/support/downloads/manuals/data_analysis_fundamentals_manual.pdf)). Additionally, the signal was transformed and per chip and per gene normalisation steps were performed in GeneSpring v6.1 prior to detailed analysis of the data: 1) signals <0.01 transformed to 0.01 to allow more efficient analysis of log transformed data; 2) per chip, each measurement on the array was normalised to the 50^th^ percentile of all measurements on the array and 3) per gene, each gene was normalised to its median value across all arrays in the experiment, to compare the relative gene expression changes of each gene in different samples.

Identification of genes whose expression is consistently and significantly altered as a consequence of chemoradiotherapy or radiotherapy: Subsequent filtering of data was performed using MicroDBv5.0 and DMTv3.1 (Affymetrix, Santa Clara, CA) and additional threshold filtering and hierarchical cluster analysis was performed using GeneSpring v6.1 *(Silicon Genetics, Redwood City, CA).* This analysis used is outlined and summarised in figure S2.Iinitial threshold filtering and then a probalistic (Welch’s t-test, p<0.01) of filtering of data. At the threshold filtering stage, genes were identified that were called ‘present’ (GCOSv1.2) in at least 1 out of 16 of the tumour specimens, and that were called increased or decreased (GCOSv1.2) in pre- versus post-tumour specimens by ≥ 1.5 fold for all (4/4) patients in each neoadjuvant treatment group. At this stage genes that were increased or decreased ≥1.5 fold in 2/2 of the untreated controls were removed, before the probabilistic filtering of data (figure S2). The threshold of a 1.5 fold increase in all pre- versus post-treatment specimens in each group was chosen based upon evidence from previous investigations, which demonstrated the reproducibility, technical accuracy (with RT-PCR, see and biological validity (protein expression and relationship to clinical outcome or cell characteristics) of this approach [ see Wang et al 2004 and Petty et al 2006 referenced in manuscript].

**Figure S2.** Schematic to illustrate bioinformatics analysis performed to identify genes whose expression was consistently and significantly altered as a result of either neoadjuvant chemoradiotherapy or short course radiotherapy. Initial threshold filtering (changed in all 4/4 **≥** 1.5 fold) was performed during which genes consistently elevated above threshold in the control no treatment group were removed and then probabilistic filtering (Welch’s t-test, p<0.01) as shown.**^1^**Present is as called by GCOS v1.2 using the detection call algorithm **^2^** Increase or Decrease is as called by GCOSv1.2 in comparative analysis pre versus post tumour specimens using the change call algorithm.
