# Supplementary material for: APRIL is a novel clinical chemo-resistance biomarker in colorectal adenocarcinoma identified by gene expression profiling
Source: BMC Cancer. 2009 Dec 11;9:434. doi: 10.1186/1471-2407-9-434 (PMC2801520; doi:10.1186/1471-2407-9-434)
Supplement: Additional file 3 — Details of genes identified in analysis of rectal adenocarcinomas. Details of genes identified in analysis of rectal adenocarcinomas whose expression is consistently and significantly changed after treatment with chemoradiotherapy or radiotherapy. Table S3.1-. List of 86 genes (91 probe sets) whose expression is consistently and significantly changed after treatment with chemoradiotherapy. Table S3.2 -List of 52 genes (58 probe sets) whose expression is consistently and significantly chaged after treatment with short course radiotherapy. [file 1471-2407-9-434-S3.DOC]

**Additional File 3**

**Table S3.1** List of 86 genes (91 probe sets) whose expression is consistently and significantly changed after treatment with chemoradiotherapy. Genes are grouped according to function as ascertained by reference to the publically available databases - GO, Genespring v6.1, Netaffx, EntrezGene, RefSeq and our own literature searches using Medline and ISI. Genes highlighted in italic script and in red have multiple functions and are present in more than one functional group.^1^ Fold change is mean fold change in post-treatment biopsies as compared to pre-treatment biopsies.^2^ ‘Others’: genes are placed in this group if they are the only representative of their functional group. Where there is more than one probe set * marks the set for which data is shown

| **Gene Title** | **Location** | **Gene symbol (HUGO)** | **Probe set ID** | **Fold Change^1^** |
| --- | --- | --- | --- | --- |
| Cell Cycle (n=16) |  |  |  |  |
| maternal embryonic leucine zipper kinase | 9p13.2 | MELK | 204825_at | 1.82 |
| ***Sterile alpha motif and leucine zipper containing kinase AZK*** | ***2q24.2*** | ***ZAK*** | ***1555259_at*** | ***2.56*** |
| ***Kruppel-like factor 6*** | ***10p15*** | ***KLF6*** | ***208961_s_at*** | ***-2.32*** |
| TPX2, microtubule-associated protein homolog (Xenopus laevis) | 20q11.2 | TPX2 | 210052_s_at | 3.43 |
| ***centromere protein F, 350/400ka (mitosin)*** | ***1q32-q41*** | ***CENPF*** | ***207828_s_at*** | ***2.52*** |
| protein tyrosine phosphatase type IVA, member 1 | 6q12 | PTP4A1 | 200731_s_at | -5.05 |
| ***CSE1 chromosome segregation 1-like (yeast)*** | ***20q13*** | ***CSE1L*** | ***201112_s_at*** | ***1.97*** |
| kinesin family member 15 | 3p21.31 | KIF15 | 219306_at | 2.19 |
| cell division cycle associated 1 | 1q23.3 | CDCA1 | 223381_at | 3.21 |
| CDC42 small effector 2 | 5q23.3 | CDC42SE2 | 1552613_s_at | -5.66 |
| ***prothymosin, alpha (gene sequence 28)*** | ***2q35-q36*** | ***PTMA*** | ***200772_x_at* 211921_x_at*** | ***1.58*** |
| ***B-cell CLL/lymphoma 10*** | ***1p22*** | ***BCL10*** | ***205263_at*** | ***-2.89*** |
| ***Suppressor of cytokine signaling 6*** | ***18q22.2*** | ***SOCS6*** | ***227542_at* and 206020_at*** | ***-3.48*** |
| ***Suppressor of cytokine signaling 7*** | ***17q12*** | ***SOCS7*** | ***228662_at*** | ***1.69*** |
| ***MCM8 minichromosome maintenance deficient 8 (S. cerevisiae)*** | ***20p12.3*** | ***MCM8*** | ***224320_s_at*** | ***2.54*** |
| ***mesoderm induction early response 1*** | ***1p31.2*** | ***MI-ER1*** | ***1555105_a_at*** | -2.2 |

Table S3.1 continued

| **Gene Title** | **Location** | **Gene symbol (HUGO)** | **Probe set ID** | **Fold Change^1^** |
| --- | --- | --- | --- | --- |
| **Transcription Regulation (n=13)** |  |  |  |  |
| ***mesoderm induction early response 1*** | ***1p31.2*** | ***MI-ER1*** | ***1555105_a_at*** | ***-2.2*** |
| nucleolar protein 5A (56kDa with KKE/D repeat) | 20p13 | NOL5A | 200875_s_at | 4.58 |
| ***prothymosin, alpha (gene sequence 28)*** | ***2q35-q36*** | ***PTMA*** | ***200772_x_at*** | ***1.58*** |
| hypothetical protein FLJ20485 | 7q22.3 | FLJ20485 | 218984_at | 2.6 |
| cytoplasmic polyadenylation  element binding protein 4 | 5q21 | CPEB4 | 224828_at | -2.64 |
| Elongation factor, RNA polymerase II, 2 | 5q15 | ELL2 | 240038_at | -3.06 |
| pleiomorphic adenoma gene-like 2 | 20q11.21 | PLAGL2 | 202925_s_at | 1.72 |

| high-mobility group box 3 | Xq28 | HMGB3 | 203744_at | 2.53 |
| --- | --- | --- | --- | --- |
| ***Kruppel-like factor 6*** | ***10p15*** | ***KLF6*** | ***208961_s_at*** | ***-2.32*** |
| B-cell CLL/lymphoma 11A (zinc finger protein) | 2p16.1 | BCL11A | 210347_s_at | 2.87 |
| small nuclear ribonucleoprotein polypeptides B and B1 | 20p13 | SNRPB | 213175_s_at | 1.78 |
| ***TGFB-induced factor 2 (TALE family homeobox)*** | ***20q11.2-q12*** | ***TGIF2*** | ***216262_s_at*** | ***2.73*** |
| ***FOS-like antigen 2*** | ***2p23.3*** | ***FOSL2*** | ***225262_at*** | ***-1.85*** |

Table S3.1 continued

| **Gene Title** | **Location** | **Gene symbol (HUGO)** | **Probe set ID** | **Fold Change^1^** |
| --- | --- | --- | --- | --- |
| Cell Death (n=12) |  |  |  |  |
| programmed cell death 4 (neoplastic transformation inhibitor) | 10q24 | PDCD4 | 1557166_at | -3.31 |
| ***prothymosin, alpha (gene sequence 28)*** | ***2q35-q36*** | ***PTMA*** | ***200772_x_at*** | ***1.58*** |
| ***B-cell CLL/lymphoma 10*** | ***1p22*** | ***BCL10*** | ***205263_at*** | ***-2.89*** |
| ***Sterile alpha motif and leucine zipper containing kinase AZK*** | ***2q24.2*** | ***ZAK*** | ***1555259_at*** | ***2.56*** |
| ***CSE1 chromosome segregation 1-like (yeast)*** | ***20q13*** | ***CSE1L*** | ***201112_s_at*** | ***1.97*** |
| ***Myeloid cell leukemia sequence 1 (BCL2-related)*** | ***1q21*** | ***MCL1*** | ***214056_at* and 200798_x_at*** | ***-3.65*** |
| ***cathepsin S*** | ***1q21*** | ***CTSS*** | ***202902_s_at*** | ***-2.99*** |
| Phosphatidylinositol-4-phosphate 5-kinase, type II, beta | 17q12 | PIP5K2B | 201080_at | 1.95 |
| ***catalase*** | ***11p13*** | ***CAT*** | ***201432_at*** | ***-1.71*** |
| ***adrenomedullin*** | ***11p15.4*** | ***ADM*** | ***202912_at*** | ***3.32*** |
| tankyrase, TRF1-interacting ankyrin-related ADP-ribose polymerase 2 | 10q23.3 | TNKS2 | 222562_s_at | -1.99 |
| ***FOS-like antigen 2*** | ***2p23.3*** | ***FOSL2*** | ***225262_at*** | ***-1.85*** |

Table S3.1 continued

| **Gene Title** | **Location** | **Gene symbol (HUGO)** | **Probe set ID** | **Fold Change^1^** |
| --- | --- | --- | --- | --- |
| **Signal Transduction (n=6)** |  |  |  |  |
| rhophilin, Rho GTPase binding protein 2 | 19q13.11 | RHPN2 | 227196_at | -3.19 |
| calcium regulated heat stable protein 1, 24kDa | 16p13.2 | CARHSP1 | 224910_at | 1.73 |
| ***Suppressor of cytokine signaling 6*** | ***18q22.2*** | ***SOCS6*** | ***227542_at*** | ***-3.49*** |
| ***Suppressor of cytokine signaling 7*** | ***17q12*** | ***SOCS7*** | ***228662_at*** | ***1.69*** |
| ***adrenomedullin*** | ***11p15.4*** | ***ADM*** | ***202912_at*** | ***-3.32*** |
| ***adenosine A2b receptor*** | ***17p12-p11.2*** | ***ADORA2B*** | ***205891_at*** | ***-2.66*** |
| **DNA Replication and Repair (n=6)** |  |  |  |  |
| topoisomerase (DNA) II alpha 170kDa | 17q21-q22 | TOP2A | 201292_at | 2.85 |
| polymerase (DNA directed), epsilon 3 (p17 subunit) | 9q33 | POLE3 | 208828_at | 1.59 |
| ***MCM8 minichromosome maintenance deficient 8 (S. cerevisiae)*** | ***20p12.3*** | ***MCM8*** | ***224320_s_at*** | ***2.54*** |
| ***centromere protein F, 350/400ka (mitosin)*** | ***1q32-q41*** | ***CENPF*** | ***207828_s_at*** | ***2.52*** |
| ***CSE1 chromosome segregation 1-like (yeast)*** | ***20q13*** | ***CSE1L*** | ***201112_s_at*** | ***1.97*** |
| RIO kinase 3 (yeast) | 18q11.2 | RIOK3 | 202129_s_at | -3.81 |

Table S3.1 continued

| **Gene Title** | **Location** | **Gene symbol (HUGO)** | **Probe set ID** | **Fold Change^1^** |
| --- | --- | --- | --- | --- |
| **Nucleotide metabolism (n=4)** |  |  |  |  |
| carbamoyl-phosphate synthetase 2, aspartate transcarbamylase, and dihydroorotase | 2p22-p21 | CAD | 202715_at | 2.59 |
| Phosphoribosylformyl  glycinamidine synthase (FGAR amidotransferase) | 17p13.1 | PFAS | 213302_at | 2.03 |
| ectonucleotide pyrophosphatase/phosphodiesterase 4 (putative function) | 6p21.1 | ENPP4 | 204160_s_at | -2.63 |
| aldehyde dehydrogenase 6 family, member A1 | 14q24.3 | ALDH6A1 | 204290_s_at | -2.51 |
| **Immune Response (n=3)** |  |  |  |  |
| ***cathepsin S*** | ***1q21*** | ***CTSS*** | ***202902_s_at*** | ***-2.99*** |
| Suppressor of cytokine signaling 6 | ***18q22.2*** | ***SOCS6*** | ***227542_at*** | ***-3.48*** |
| ***adenosine A2b receptor*** | ***17p12-p11.2*** | ***ADORA2B*** | ***205891_at*** | ***-2.66*** |
| Oxidative Stress Response (n=3) |  |  |  |  |
| ***catalase*** | ***11p13*** | ***CAT*** | ***201432_at*** | ***-1.71*** |
| ***prothymosin, alpha (gene sequence 28)*** | ***2q35-q36*** | ***PTMA*** | ***200772_x_at*** | ***1.58*** |
| ***adenosine A2b receptor*** | ***17p12-p11.2*** | ***ADORA2B*** | ***205891_at*** | ***-2.66*** |
| **Cell Adhesion (n=3)** |  |  |  |  |
| ***integrin, alpha 6*** | ***2q31.1*** | ***ITGA6*** | ***201656_at*** | ***-2.49*** |
| villin 2 (ezrin) | 6q25.2-q26 | VIL2 | 208622_s_at | -3.04 |
| desmocollin 2 | 18q12.1 | DSC2 | 226817_at | -3.54 |

Table S3.1 continued

| **Gene Title** | **Location** | **Gene symbol (HUGO)** | **Probe set ID** | **Fold Change^1^** |
| --- | --- | --- | --- | --- |
| **Angiogenesis (n=2)** |  |  |  |  |
| ***integrin, alpha 6*** | ***2q31.1*** | ***ITGA6*** | ***201656_at*** | ***-2.49*** |
| ***adenosine A2b receptor*** | ***17p12-p11.2*** | ***ADORA2B*** | ***205891_at*** | ***-2.66*** |
| **Others^2^** |  |  |  |  |
| nucleolar protein 5A (56kDa with KKE/D repeat) | 20p13 | NOL5A | 200875_s_at | 4.58 |
| enolase 2 (gamma, neuronal) | 12p13 | ENO2 | 201313_at | 2.81 |
| UDP-N-acetyl-alpha-D-galactosamine: polypeptide N -acetylgalactosaminyltransferase 1 (GalNAc-T1) | 18q12.1 | GALNT1 | 201722_s_at | -2.21 |
| cytochrome P450, family 2, subfamily B, polypeptide 7 pseudogene 1 | 19q13.2 | CYP2B7P1 | 206754_s_at | 4.42 |
| solute carrier family 4, sodium bicarbonate cotransporter, member 4 | 4q21 | SLC4A4 | 210738_s_at | -32.28 |
| fucosyltransferase 6 (alpha (1,3) fucosyltransferase) | 19p13.3 | FUT6 | 211885_x_at | -2.36 |
| nucleoporin 210kDa | 3p25.2-p25.1 | NUP210 | 213947_s_at | 2.83 |
| plexin A1 | 3q21.3 | PLXNA1 | 221538_s_at | 2.66 |
| mitochondrial ribosomal protein S26 | 20p13 | MRPS26 | 225196_s_at | 2.07 |
| Golgi autoantigen, golgin subfamily a, 1 | 9q33.3 | GOLGA1 | 228174_at | 1.71 |
| CDW92 antigen | 9q31.2 | CDW92 | 228485_s_at | -2.01 |
| ATPase family, AAA domain containing 2 | 8q24.13 | ATAD2 | 235266_at | 2.3 |
| T-cell activation protein phosphatase 2C | 12q24.11 | TA-PP2C | 235744_at | -2.82 |

Table S3.1 continued

| **Gene Title** | **Location** | **Gene symbol (HUGO)** | **Probe set ID** | **Fold Change^1^** |
| --- | --- | --- | --- | --- |
| **Unknown Function** |  |  |  |  |
| family with sequence similarity 55, member A | 11q23.2 | FAM55A | 1561387_a_at | 4.42 |
| KIAA0494 gene product | 1pter-p22.1 | KIAA0494 | 201776_s_at | -2.03 |
| transmembrane protease, serine 2 | 21q22.3 | TMPRSS2 | 205102_at* and 211689_s_at | -3.43 |
| KIAA0186 gene product | 20p11.21 | KIAA0186 | 206102_at | 3.51 |
| LAS1-like (S. cerevisiae) | Xq12-q13 | LAS1L | 208117_s_at | 1.96 |
| transmembrane emp24 protein transport domain containing 7 | 5q22.3 | TMED7 | 209404_s_at | -2.75 |
| transmembrane protease, serine 2 | 21q22.3 | TMPRSS2 | 211689_s_at | -8.76 |
| hypothetical protein FLJ10719 | 15q25-q26 | FLJ10719 | 213008_at* and 213007_at | 3.66 |
| fem-1 homolog c (C.elegans) | 5q22 | FEM1C | 213341_at | -2.43 |
| KIAA0828 protein | 7q32.3 | KIAA0828 | 215672_s_at | 4.73 |
| similar to prothymosin alpha | 12p13.2 | LOC440085 | 216384_x_at | 2.52 |
| ubiquitin-conjugating enzyme E2, J1 (UBC6 homolog, yeast) | 6q15 | UBE2J1 | 217826_s_at | 2.05 |
| hypothetical protein FLJ20485 | 7q22.3 | FLJ20485 | 218984_at | 2.6 |
| hypothetical protein FLJ21272 | 1q21.2 | FLJ21272 | 220467_at | 2.78 |
| hypothetical protein FLJ22104 | 11q14.2 | FLJ22104 | 222209_s_at | 1.98 |
| family with sequence similarity 29, member A | 9p22.1 | FAM29A | 222685_at | 2.17 |
| SCY1-like 2 (S. cerevisiae) | 12q23.1 | SCYL2 | 224960_at | -2.29 |

Table S3.1 continued

| **Gene Title** | **Location** | **Gene symbol (HUGO)** | **Probe set ID** | **Fold Change^1^** |
| --- | --- | --- | --- | --- |
| **Unknown Function** (continued) |  |  |  |  |
| Hypothetical protein LOC286144 | 8q22.1 | LOC286144 | 225599_s_at | -2.47 |
| disrupted in renal carcinoma 2 | 3q21.1 | DIRC2 | 226026_at | -2.25 |
| FYVE, RhoGEF and PH domain containing 5 | 3p25.1 | FGD5 | 226985_at | 2.72 |
| Transcribed locus | NK | Not named | 227921_at | 3.68 |
| Hypothetical protein FLJ14503 | Xp22.12 | FLJ14503 | 228262_at | 12.94 |
| Hypothetical protein FLJ11029 | 17q23.2 | FLJ11029 | 228273_at | 2.92 |
| Transcribed locus | NK | Not named | 230312_at | 5.04 |
| hypothetical protein LOC285074 | 2p11.1 | LOC285074 | 230588_s_at | 2.19 |
| KIAA1274 | 10q22.1 | KIAA1274 | 231887_s_at | 3.32 |
| KIAA1913 | 6q23.1 | KIAA1913 | 234994_at | -2.62 |
| shugoshin-like 2 (S. pombe) | 2q33.1 | SGOL2 | 235425_at | 2.7 |

**Table S3.2** List of 52 genes (58 probe sets) whose expression is consistently and significantly changed after treatment with short course radiotherapy. Genes are grouped according to function as ascertained by reference to the publically available databases - GO, Genespring v6.1, Netaffx, EntrezGene, RefSeq and our own literature searches using Medline and ISI. Genes highlighted in italic script and in red have multiple functions and are present in more than one functional group.^1^ Fold change is mean fold change in post-treatment biopsies as compared to pre-treatment biopsies.^2^ ‘Others’: genes are placed in this group if they are the only representative of their functional group although angiogenesis and cell adhesion have been highlighted due to the known importance in radiotherapy response. Where there is more than 1 probe set * marks probe set for which data is shown

| **Gene Title** | **Gene Symbol (HUGO)** | **Location** | **Probe Set ID** | **Fold Change^1^** |
| --- | --- | --- | --- | --- |
| **Oxidative Stress Response (n=4)** |  |  |  |  |
| ***fibroblast growth factor 7 (keratinocyte growth factor)*** | ***FGF7*** | ***15q15-q21.1*** | ***1554741_s_at*** | ***-5.63*** |
| ***N-myc downstream regulated gene 1*** | ***NDRG1*** | ***8q24.3*** | ***200632_s_at*** | ***2.02*** |
| similar to RIKEN cDNA 2310016C16 | LOC493869 | 5q11.2 | 227628_at | -2.91 |
| succinate dehydrogenase complex, subunit A, flavoprotein (Fp) | SDHA | 5p15 | 201093_x_at* 222021_x_at | 1.87 |
| **Transcription Regulation (n=4)** |  |  |  |  |
| ***nuclear factor I/B*** | ***NFIB*** | ***9p24.1*** | ***209290_s_at*** | ***1.78*** |
| cAMP responsive element modulator | CREM | 10p11.21 | 209967_s_at | -6.93 |
| elongation factor RNA polymerase II-like 3 | ELL3 | 15q15.3 | 219517_at | 2.98 |
| cyclin M3 | CNNM3 | 2p12-p11.2 | 220739_s_at | 2.05 |
| **Cell Death (n=3)** |  |  |  |  |
| ***fibroblast growth factor 7 (keratinocyte growth factor)*** | ***FGF7*** | ***15q15-q21.1 /*** | ***1554741_s_at*** | ***-5.63*** |
| ***N-myc downstream regulated gene 1*** | ***NDRG1*** | ***8q24.3*** | ***200632_s_at*** | ***2.02*** |
| serine/threonine kinase 17a (apoptosis-inducing) | STK17A | 7p12-p14 | 202693_s_at | -2.09 |
| Cell Cycle (n=3) |  |  |  |  |
| ***fibroblast growth factor 7 (keratinocyte growth factor)*** | ***FGF7*** | ***15q15-q21.1*** | ***1554741_s_at*** | ***-5.63*** |
| cyclin-dependent kinase inhibitor 1A (p21, Cip1) | CDKN1A | 6p21.2 | 202284_s_at | 3.57 |
| decorin | DCN | 12q21.33 | 209335_at | -3.47 |

Table S3.2 continued

| **Gene Title** | **Gene Symbol (HUGO)** | **Location** | **Probe Set ID** | **Fold Change^1^** |
| --- | --- | --- | --- | --- |
| **Signal Transduction (n=2)** |  |  |  |  |
| phosphodiesterase 1A, calmodulin-dependent | PDE1A | 2q32.1 | 1558680_s_at | -2.86 |
| Proprotein convertase subtilisin/kexin type 6 | PCSK6 | 15q26 | 242662_at | -2.89 |
| **DNA replication and Repair (n=2)** |  |  |  |  |
| ***nuclear factor I/B*** | ***NFIB*** | ***9p24.1*** | ***209290_s_at*** | ***1.78*** |
| ligase IV, DNA, ATP-dependent | LIG4 | 13q33-q34 | 227766_at | -1.75 |
| **Angiogenesis (n=1)** |  |  |  |  |
| matrix metalloproteinase 19 | MMP19 | 12q14 | 204575_s_at | -3.96 |
| **Cell Adhesion (n=1)** |  |  |  |  |
| p21/Cdc42/Rac1-activated kinase 1 (STE20 homolog, yeast) | PAK1 | 11q13-q14 | 226507_at | 1.91 |
| **Other^2^ (n=11)** |  |  |  |  |
| stomatin | STOM | 9q34.1 | 201061_s_at* | -2.46 |
| AFG3 ATPase family gene 3-like 2 (yeast) | AFG3L2 | 18p11 | 202486_at | 1.7 |
| protease, serine, 16 (thymus) | PRSS16 | 6p21 | 208165_s_at | 3.36 |
| PTPRF interacting protein, binding protein 2 (liprin beta 2) | PPFIBP2 | 11p15.4 | 212841_s_at | 2.14 |
| pyridoxal (pyridoxine, vitamin B6) kinase | PDXK | 21q22.3 | 218019_s_at | 2.55 |
| FK506 binding protein 14, 22 kDa | FKBP14 | 7p15.1 | 219390_at | -2.92 |
| FK506 binding protein 7 | FKBP7 | 2q31.2 | 224002_s_at | -2.13 |
| sulfatase 2 | SULF2 | 20q12-q13.2 | 224724_at | -1.81 |
| pregnancy-associated plasma protein A, pappalysin 1 | PAPPA | 9q33.2 | 224941_at* 224940_s_at 224942_at 228128_x_at 232728_at | -4.29 |

Table S3.2 continued

| **Gene Title** | **Gene Symbol (HUGO)** | **Location** | **Probe Set ID** | **Fold Change^1^** |
| --- | --- | --- | --- | --- |
| serine protease inhibitor, Kunitz type 1 | SPINT1 | 15q15.1 | 202826_at | 1.97 |
| calcium/calmodulin-dependent protein kinase II | CaMKIINalpha | 1p36.12 | 218309_at | 2.38 |
| Unknown (n=24) |  |  |  |  |
| armadillo repeat containing, X-linked 2 | ARMCX2 | Xq21.33-q22.2 | 203404_at | -2.72 |
| GLI pathogenesis-related 1 (glioma) | GLIPR1 | 12q21.1 | 204221_x_at* 201060_x_at | -2.99 |
| lung type-I cell membrane-associated glycoprotein | T1A-2 | 1p36.21 | 204879_at | -3.62 |
| hippocalcin-like 1 | HPCAL1 | 2p25.1 | 212552_at | 1.88 |
| calcium/calmodulin-dependent protein kinase II | CaMKIINalpha | 1p36.12 | 218309_at | 2.38 |
| armadillo repeat containing, X-linked 1 | ARMCX1 | Xq21.33-q22.2 | 218694_at | -2.45 |
| motile sperm domain containing 1 | MOSPD1 | Xq26.3 | 218853_s_at | -1.94 |
| squamous cell carcinoma antigen recognized by T cells 2 | SART2 | 6q22 | 218854_at | -3.84 |
| single Ig IL-1R-related molecule | SIGIRR | 11p15.5 | 218921_at | 1.56 |
| hypothetical protein FLJ22833 | FLJ22833 | 2q32.3 | 222872_x_at | -3.21 |
| LOC440448 | Not named | 17q23.2 | 224806_at | 1.89 |
| prickle-like 2 (Drosophila) | PRICKLE2 | 3p14.1 | 225968_at | -2.41 |
| transcribed locus | Not named | NK | 226136_at | -2.62 |
| Similar to ankyrin-repeat protein Nrarp | MGC61598 | 9q34.3 | 226499_at | 3.54 |
| Homo sapiens, clone IMAGE:5261213, mRNA | Not named | NK | 227221_at | -4.71 |
| leucine-rich repeats and calponin homology (CH) domain containing 2 | LRCH2 | Xq23 | 227688_at | -3.12 |
| nucleosome assembly protein 1-like 5 | NAP1L5 | 4q22.1 | 228063_s_at | -2.76 |

Table S3.2 continued

| **Gene Title** | **Gene Symbol (HUGO)** | **Location** | **Probe Set ID** | **Fold Change^1^** |
| --- | --- | --- | --- | --- |
| Unknown  (continued) |  |  |  |  |
| LOC132671 | LOC132671 | 4q11 | 229331_at | -4.42 |
| Transcribed locus | Not named | NK | 230653_at | 2.88 |
| Transcribed locus | Not named | NK | 230728_at | -1.96 |
| hypothetical protein FLJ22833 | FLJ22833 | 2q32.3 | 233085_s_at | -3.02 |
| Transcribed locus, moderately similar to NP_060312.1 hypothetical protein FLJ20489 [Homo sapiens] | Not named | NK | 239066_at | 2.73 |
| hypothetical protein LOC151878 | LOC151878 | 3p14.3 | 239965_at | 2.92 |
| Similar to chromosome 15 open reading frame 16; cezanne 2 | Not named | 15q13.2 | 240247_at | 2.84 |
| ELISC-1 | Not named | NK | 244354_at | 3.17 |
