# Supplementary material for: APRIL is a novel clinical chemo-resistance biomarker in colorectal adenocarcinoma identified by gene expression profiling
Source: BMC Cancer. 2009 Dec 11;9:434. doi: 10.1186/1471-2407-9-434 (PMC2801520; doi:10.1186/1471-2407-9-434)
Supplement: Additional file 4 — Biological pathways altered following neoadjuvant radiotherapy or chemoradiotherapy in rectal tumours. The number of genes in each biological pathway whose expression was altered following chemoradiotherapy or radiotherapy is shown. Gene ontologies (biological function) were assigned according to GO, Genespring v6.1, Netaffx, EntrezGene, RefSeq and literature searches using Medline and ISI. Table S4 - The number of genes in each biological pathway whose expression was altered following chemoradiotherapy or radiotherapy. [file 1471-2407-9-434-S4.DOC]

**Additional file 4**

Table S4 Biological pathways altered following neoadjuvant radiotherapy or chemoradiotherapy in rectal tumours.

| **Gene function^1^** | **Chemoradiotherapy**  **(n=86)^2^** | **Short Course Radiotherapy (n=51)^3^** | **P value^4^** |
| --- | --- | --- | --- |
| Cell Cycle (n=1164) | 16 (19%) | 3 (6%) | **0.003** |
| Cell Death (n=2177) | 12 (14%) | 3 (6%) | **0.020** |
| Oxidative Stress (n=1037) | 3 (3%) | 4 (8%) | 0.705 |
| Transcriptional regulation (n=20807) | 13 (15%) | 4 (8%) | **0.029** |
| Signal Transduction (n=14858) | 6 (7%) | 2 (4%) | 0.157 |
| DNA Replication & Repair (n=2426) | 6 (7%) | 2 (4%) | 0.157 |
| Cell Adhesion (n=7096) | 3 (3%) | 1 (2%) | 0.317 |
| Angiogenesis (n=447) | 2 (2%) | 1 (2%) | 0.563 |
| Pyrimidine Metabolism (n=131) | 4 (5%) | 0 (0%) | **0.044** |
| Immune response (n=2991) | 3 (3%) | 0 (0%) | 0.083 |
| Others where n=1 | 13 (15%) | 12 (24%) | N/A |
| Unknown function | 26 (30%) | 24 (47%) | N/A |

^1^ Genes are grouped according to biological function. Gene ontologies (biological function) were assigned according to GO, Genespring v6.1, Netaffx, EntrezGene, RefSeq and literature searches using Medline and ISI. Each gene may be classified into more than one functional group (further details in *supplementary tables 1 and 2*).

The number of genes in each biological pathway whose expression was altered following chemoradiotherapy^2^ or radiotherapy^3^ is shown (percentage of total number of genes altered after treatment [chemoradiotherapy total = 86 genes and radiotherapy total = 51 genes] is shown in parentheses).

Chi-square test for significance is shown^4^, where the number of probe sets in a specific biological pathway^1^ altered after treatment with either chemoradiotherapy^2^ or radiotherapy^3^, relative to the total number of probe sets representing that biological pathway on the HGU133 Plus 2.0 microarray was analysed.
