# Supplementary material for: APRIL is a novel clinical chemo-resistance biomarker in colorectal adenocarcinoma identified by gene expression profiling
Source: BMC Cancer. 2009 Dec 11;9:434. doi: 10.1186/1471-2407-9-434 (PMC2801520; doi:10.1186/1471-2407-9-434)
Supplement: Additional file 6 — Details of Supervised analysis of Cell Death Pathways. Schematic representations to explain supervised bionformatic analysis of cell death pathways. Figure S6 Schematic illustrating the bioinformatics analysis performed for the supervised analysis of cell death genes. GCOSv1.2 and Genespring v6.1 were used for the analyses. Table S6. List of Cell death genes identified in this analysis in CRT and SCRT treated rectal adenocarcinoma patients. Table S6.1. Genes identified as candidate novel mechanisms of 5FU chemoresistance or sensitivity from gene expression profiling experiments of 5FU resistant colorectal and breast cancer cell lines [file 1471-2407-9-434-S6.DOC]

**Additional File 6**

**Supervised analysis of Cell Death Pathways**

We identified 2177 genes (see Supplementary information 5) involved in the control, regulation and execution of cell death( apoptotic and non-apoptotic forms) that were represented on the HGU133 Plus 2.0 GeneGhip, using databases (GO, Genespring v6.1, RefSeq, EntrezGene) and literature searches (Medline and ISI). Subsequent analysis using this involved threshold and probalistic filtering of data as described ( supplementary information 2). Figure S6 outlines and summarises the analysis and Table S6 shows the genes indentified

**Figure S5.** Schematic illustrating the bioinformatics analysis performed for the supervised analysis of cell death genes. GCOSv1.2 and Genespring v6.1 were used for the analyses.

**Table S6 : Cell death genes altered in rectal adenocarcinomas following chemoradiotherapy or radiotherapy**. (a) Genes whose expression was consistently and significantly altered in rectal tumours following chemoradiotherapy or (b) radiotherapy in a supervised analysis of 2177 cell death genes (see supplementary information 4) represented on the HGU133 Plus 2.0 microarray (Affymetrix, CA).

^1^ Fold change is mean normalised signal in post-treatment tumour biopsies/ mean normalised signal in pre-treatment biopsies.

* Denotes probe set for which data is shown where more than one probe set for a specific gene was identified in the analysis.

(a) (a)

(b) (a)

| (a) Gene Title | Gene Symbol (HUGO) | Location | Probe Set ID | Fold Change^1^ |
| --- | --- | --- | --- | --- |
| programmed cell death 4 (neoplastic transformation inhibitor) | PDCD4 | 10q24 | 1557166_at*  202730_s_at  202731_at | 3.31 |
| myeloid cell leukemia sequence 1 (BCL2-related) | MCL1 | 1q21 | 200798_x_at | 4.65 |
| testis enhanced gene transcript (BAX inhibitor 1) | TEGT | 12q12-q13 | 200803_s_at | 1.88 |
| Phosphatidylinositol-4-phosphate 5-kinase, type II, beta | PIP5K2B | 17q12 | 201080_at | -2.00 |
| CSE1 chromosome segregation 1-like (yeast) | CSE1L | 20q13 | 201112_s_at | -2.00 |
| forkhead box O1A (rhabdomyosarcoma) | FOXO1A | 13q14.1 | 202724_s_at | 2.11 |
| cathepsin S | CTSS | 1q21 | 202902_s_at | 2.99 |
| B-cell CLL/lymphoma 11 | BCL10 | 1p22 | 205263_at | 2.89 |
| copine I | CPNE1 | 20q11.22 | 206918_s_at | -1.82 |
| HIV-1 Tat interactive protein 2, 30kDa | HTATIP2 | 11p15.1 | 207180_s_at | 1.99 |
| tumor necrosis factor (ligand) superfamily, member 13 (APRIL) | TNFSF13 | 17p13.1 | 209499_x_at | 2.85 |
| tribbles homolog 3 (Drosophila) | TRIB3 | 20p13-p12.2 | 218145_at | -5.56 |
| kinesin family member 20A | KIF20A | 5q31 | 218755_at | -2.22 |
| catenin, beta like 1 | CTNNBL1 | 20q11.23-q12 | 221021_s_at | -1.56 |
| tankyrase, TRF1-interacting ankyrin-related ADP-ribose polymerase 3 | TNKS2 | 10q23.3 | 222562_s_at | 1.99 |
| sphingosine-1-phosphate phosphatase 2 | SGPP1 | 14q23.2 | 223391_at | 2.91 |
| FOS-like antigen 3 | FOSL2 | 2p23.3 | 225262_at | 1.85 |

| (b) Gene Title | Gene Symbol (HUGO) | Location | Probe set ID | Fold Change1 |
| --- | --- | --- | --- | --- |
| BCL2-like 11 (apoptosis facilitator) | BCL2L11 | 2q13 | 1555372_at | -1.52 |
| cathepsin K (pycnodysostosis) | CTSK | 1q21 | 202450_s_at | 3.35 |
| serine/threonine kinase 17a (apoptosis-inducing) | STK17A | 7p12-p14 | 202693_s_at | 2.09 |
| Cystatin A (stefin A) | CSTA | 3q21 | 204971_at | 2.14 |
| paternally expressed 3 | PEG3 | 19q13.4 | 209242_at | 3.41 |
| cell division cycle 2-like 6 (CDK8-like) | CDC2L6 | 6q21 | 212897_at | -1.52 |
| Mdm2, transformed 3T3 cell double minute 2, p53 binding protein (mouse) | MDM2 | 12q14.3-q15 | 217373_x_at | 14.32 |
| engulfment and cell motility 3 (ced-12 homolog, C. elegans) | ELMO3 | 16q22.1 | 219411_at | -2.70 |
| apoptosis-related protein PNAS-1 | FLJ39616 | 12q24.12 | 64432_at | -2.24 |

This analysis of cell death genes implicates several distinct pathways as important molecular determinants of clinical response to either chemoradiotherapy or short course radiotherapy. In the chemoradiotherapy treated patients the NFκB pathway is implicated by the increase in post treatment tumour specimens of 4 genes involved in NFκB mediated cell survival signalling; BCL10, CPNE1, APRIL/TNFSF13 and TRIB3[Kern et al., 2004;Lucas et al., 2004;Tomsig et al., 2004;Wu et al., 2003]. The pattern of change in expression of these genes after chemoradiotherapy is consistent with activation of NFkB signaling, (CPNE1 and TRIB3 are both ‘antagonists’ of NFkB function and are decreased; BCL10 and APRIL/TNFSF13 are both upstream activators of NFkB signaling and are increased).

Similarly, 3 genes from or interacting with the BCL2 family (MCL1, TEGT1 and HTATIP2) have increased expression in the post chemoradiotherapy specimens, implicating the potential importance of this family of proteins and there interrelated pathways and perhaps regulation of mitochondrial membrane permeability in general[Grzmil et al., 2003;Huckelhoven, 2004;Xiao et al., 2000;Xu and Reed, 1998]. Another cell member of the BCL2 family, BCL2L is increased post-radiotherapy.

These data from CRT or RT treated tumours are consistent with reports implicating NFkB activation in 5FU resistance and of the importance of BCL2 family members (including MCL1 identified in the analysis here), in the clinical response to chemoradiotherapy in rectal adenocarcinoma and in the cellular response to ionising radiation[Chang et al., 2005;Kim et al., 2001;Lee et al., 1999;Mackey et al., 1998;Nix et al., 2005;Scopa et al., 2001;Streffer et al., 2002].

Two molecules identified in the chemoradiotherapy group PIP5KB and CSE1L have been implicated as downstream effectors of TNF induced apoptosis, interestingly both are downregulated after treatment.

The lysosomal cysteine protease cathepsin S is increased in the post-chemoradiotherapy specimens. Additionally, cathepsin K and the lysosomal protease inhibitor cystatin A, are increased post-radiotherapy. This may implicate the lysosomal cell death pathways as a mechanism of action for both treatment types.

In post radiotherapy treated specimens, MDM2 is 14 fold increased. MDM2 has been implicated in radioresistance in both cell line and clinical studies for several different human tumour types, but not previously rectal adenocarcinoma [Perry, 2004]. Accordingly, our data implicate it as potentially important for rectal adenocarcinomas treated with RT.

TNKS2 is a member of the PARP family (alias PARP5a) that has been shown to bring about a necrotic-like programmed cell death [Kaminker et al., 2001]. We found it to be increased in post-chemoradiotherapy treated specimens.

Overall, in both chemoradiotherapy and short course treated rectal adenocarcinomas, the transcriptional analysis of cell death pathways portrays a picture of the importance of multiple and distinct cell death pathways in determination of clinical response. These include both apoptotic and non-apoptotic pathways. The Bcl-2 family of proteins (and the mitochondrion) and their related pathways and also NFκB represent key regulatory points for several different cell death pathways and our analysis suggests that they may be important in this role in clinical response determination (see figure 6.3.8 and 6.3.9).

The importance of multiple cell death pathways contrasts with our gene expression analysis of NSCLC, where the data suggested a more limited involvement of distinct cell death pathways in clinical response determination. This may have important implications for novel therapeutic strategies targeting these two malignancies.

**REFERENCES**

Chang HJ, Jung KH, Kim DY, Jeong SY, Choi HS, Kim YH, Sohn DK, Yoo BC, Lim SB, Kim DH, Ahn JB, Kim IJ, Kim JM, Yoon WH, Park JG (2005) Bax, a predictive marker for therapeutic response to preoperative chemoradiotherapy in patients with rectal carcinoma. *Hum Pathol* 36: 364-371

Grzmil M, Thelen P, Hemmerlein B, Schweyer S, Voigt S, Mury D, Burfeind P (2003) Bax inhibitor-1 is overexpressed in prostate cancer and its specific down-regulation by RNA interference leads to cell death in human prostate carcinoma cells. *Am J Pathol* 163: 543-552

Huckelhoven R (2004) BAX Inhibitor-1, an ancient cell death suppressor in animals and plants with prokaryotic relatives. *Apoptosis* 9: 299-307

Kaminker PG, Kim SH, Taylor RD, Zebarjadian Y, Funk WD, Morin GB, Yaswen P, Campisi J (2001) TANK2, a new TRF1-associated poly(ADP-ribose) polymerase, causes rapid induction of cell death upon overexpression. *J Biol Chem* 276: 35891-35899

Kern C, Cornuel JF, Billard C, Tang R, Rouillard D, Stenou V, Defrance T, Ajchenbaum-Cymbalista F, Simonin PY, Feldblum S, Kolb JP (2004) Involvement of BAFF and APRIL in the resistance to apoptosis of B-CLL through an autocrine pathway. *Blood* 103: 679.

Kim NK, Park JK, Lee KY, Yang WI, Yun SH, Sung J, Min JS (2001) p53, BCL-2, and Ki-67 expression according to tumor response after concurrent chemoradiotherapy for advanced rectal cancer. *Ann Surg Oncol* 8: 418-424

Lee JU, Hosotani R, Wada M, Doi R, Kosiba T, Fujimoto K, Miyamoto Y, Tsuji S, Nakajima S, Nishimura Y, Imamura M (1999) Role of Bcl-2 family proteins (Bax, Bcl-2 and Bcl-X) on cellular susceptibility to radiation in pancreatic cancer cells. *Eur J Cancer* 35: 1374-1380

Lucas PC, McAllister-Lucas LM, Nunez G (2004) NF-kappaB signaling in lymphocytes: a new cast of characters. Mackey TJ, Borkowski A, Amin P, Jacobs SC, Kyprianou N (1998) bcl-2/bax ratio as a predictive marker for therapeutic response to radiotherapy in patients with prostate cancer. *Urology* 52: 1085-1090

Mackey TJ, Borkowski A, Amin P, Jacobs SC, Kyprianou N (1998) bcl-2/bax ratio as a predictive marker for therapeutic response to radiotherapy in patients with prostate cancer. *Urology* 52: 1085-1090

Nix P, Cawkwell L, Patmore H, Greenman J, Stafford N (2005) Bcl-2 expression predicts radiotherapy failure in laryngeal cancer. *Br J Cancer* 92: 2185-2189

Perry ME (2004) Mdm2 in the response to radiation. *Mol Cancer Res* 2: 9-19

Scopa CD, Vagianos C, Kardamakis D, Kourelis TG, Kalofonos HP, Tsamandas AC (2001) bcl-2/bax ratio as a predictive marker for therapeutic response to radiotherapy in patients with rectal cancer. *Appl Immunohistochem Mol Morphol* 9: 329-334

Streffer JR, Rimner A, Rieger J, Naumann U, Rodemann HP, Weller M (2002) BCL-2 family proteins modulate radiosensitivity in human malignant glioma cells. *J Neurooncol* 56: 43-49

Tomsig JL, Sohma H, Creutz CE (2004) Calcium-dependent regulation of tumour necrosis factor-alpha receptor signalling by copine. *Biochem J* 378: 1089-1094

Wu Y, Bressette D, Carrell JA, Kaufman T, Feng P, Taylor K, Gan Y, Cho YH, Garcia AD, Gollatz E, Dimke D, LaFleur D, Migone TS, Nardelli B, Wei P, Ruben SM, Ullrich SJ, Olsen HS, Kanakaraj P, Moore PA, Baker KP (2000) Tumor necrosis factor (TNF) receptor superfamily member TACI is a high affinity receptor for TNF family members APRIL and BLyS. *J Biol Chem* 275: 35478-35485

Xu Q, Reed JC (1998) Bax inhibitor-1, a mammalian apoptosis suppressor identified by functional screening in yeast. *Mol Cell* 1: 337-346

Xu R, Wunsch D (2005) Survey of clustering algorithms. *IEEE Trans Neural Netw* 16: 645-678

**Table S6.1** Genes identified as candidate novel mechanisms of 5FU chemoresistance or sensitivity from gene expression profiling experiments of 5FU resistant colorectal and breast cancer cell lines [11] and studies of pre- and post-chemoradiotherapy treated rectal adenocacinomas, performed by our laboratory using the same profiling technology ( Affymetrix GeneChip system, HGU133A microarrays for cell line work and HGU133Plus2.0 for rectal adenocarcinomas) and same bioinformatics analysis to identify genes whose expression is consistently and significantly altered. All 4 genes show the same direction of fold change in each experiment with the exception of FLJ10719. This may be a consequence of the different probe set identified in each analysis, but may also reflect expression change consistent with response or sensitivity in the post-rectal adenocarcinoma specimens. APRIL has been selected for further validation as a putative novel 5FU chemoresiatnce mechanism, predictive biomarker and novel target as outlined in the main manuscript text.

| Gene title and symbol | Probe set i.d. | Fold Change 5FU resistant cell lines | Fold change post chemoradiotherapy in rectal adenocarcinomas |
| --- | --- | --- | --- |
| Tumour necrosis factor (ligand) superfamily, member 13 TNFSF13 (APRIL) | 209499_x_at | 1.90 | 2.85 |
| Bloom Syndrome BLM | 205733_at | -1.89 | -2.05 |
| RAD54 homolog B (S. cerevisiae) RAD54B | 219494_at | -2.48 | -3.09 |
| Hypothetical protein FLJ10719 | 213007_at and 213008_at | -3.66 | 1.83 |
