# Supplementary material for: APRIL is a novel clinical chemo-resistance biomarker in colorectal adenocarcinoma identified by gene expression profiling
Source: BMC Cancer. 2009 Dec 11;9:434. doi: 10.1186/1471-2407-9-434 (PMC2801520; doi:10.1186/1471-2407-9-434)
Supplement: Additional file 7 — Additional survival analyses for APRIL protein expression in colorectal adenocarcinomas. Kaplan-Meier survival plots for APRIL protein expression in tumour cells of colorectal adenocarcinom patients in stage I, II and II and APRIl stroma expression in Stage I and II Figure S7.1 Kaplan-Meier survival plots for APRIL immunohistochemistry showing no significant relationship for tumour cell protein expression and survival. All patients (n = 234), analysed according to intensity of APRIL staining in tumour cells [weak, moderate or strong (b)] or positive versus negative tumour cell staining (a), or stratified according to stage Dukes A/Stage I, Dukes B/Stage II and Dukes C/Stage III (c). Figure S7.2. Kaplan-Meier survival plots for APRIL immuno-histochemistry showing that positive staining in the tumour stroma shows no association with survival in Duke's A/Stage I (n = 46) or B/Stage II tumours (n = 86). [file 1471-2407-9-434-S7.DOC]

**Additional File 7**

**Figure S7.1** Kaplan-Meier survival plots for APRIL immunohistochemistry showing no significant relationship for tumour cell protein expression and survival. All patients (n=234), analysed according to intensity of staining, APRIL tumour cell weak, moderate or strong (b), positive versus negative tumour cell staining (a), or stratified according to stage Dukes A/Stage I, Dukes B/Stage II and Dukes C/Stage III (c).

**Figure S7.2.** Kaplan-Meier survival plots for APRIL immuno-histochemistry showing that positive staining in the tumour stroma shows

no association with survival in Duke’s A/Stage I (n=46) or B/Stage II tumours (n=86)
